# Endothelial cells LEENE on noncoding RNAs in diabetic vasculopathy

**DOI:** 10.1172/JCI167047

**Published:** 2023-02-01

**Authors:** Aneesh Kallapur, Tamer Sallam

**Affiliations:** 1 Division of Cardiology, Department of Medicine,; 2 Department of Physiology, and; 3 Molecular Biology Institute, UCLA, Los Angeles, California, USA.

## Abstract

Long noncoding RNAs (lncRNAs) have emerged as key mediators of regulated gene expression in diverse biologic contexts, including cardiovascular disease. In this issue of the *JCI*, Tang, Luo, and colleagues explored the contributions of lncRNAs in diabetic vasculopathy. The authors identified the lncRNA LEENE as a key mediator of angiogenesis and ischemic response. In a model of diabetic peripheral arterial disease, loss of LEENE led to impaired vascular perfusion, while its overexpression rescued the ischemic defect. The authors used unbiased chromatin affinity assays to decipher LEENE’s interactome and mode of action. These findings offer insights as to why patients with diabetes are uniquely susceptible to developing peripheral vascular disease and fill important gaps in our understanding of mechanisms that connect metabolic dysregulation with impaired angiogenesis.

## The intersection of vascular biology and noncoding genes

The 1980s marked a number of seminal discoveries in vascular biology, including Furchgott’s description of endothelium-dependent vessel relaxation mediated by what was later identified as nitric oxide (1), the discovery of the angiogenic factor VEGF (2), and the first description of angiogenesis in vitro (3). During the same era, the notion that the vast majority of DNA was made of so-called “selfish” elements was highly popularized in reference to the fact that most genetic material in higher organisms does not code for proteins and was thought to be only essential for its own self-preservation (4). While the ensuing decades witnessed an explosion in studies detailing the mechanisms and regulatory circuits controlling angiogenesis, there was very little traction in counteracting the established dogma that the majority of DNA is useless. That was the case until evidence from the Human Genome Project surprisingly showed that pervasive transcription from some of the selfish elements is highly common. Galvanized by this observation, researchers subsequently produced multiple lines of evidence suggesting that at least a subset of transcripts greater than 200 bp, which do not code for protein, participate in diverse biologic processes and are essential for life (5). Collectively, these transcripts are referred to today as long noncoding RNAs (lncRNAs).

## Integration of metabolic abnormalities with angiogenic responses

**Figure 1:**
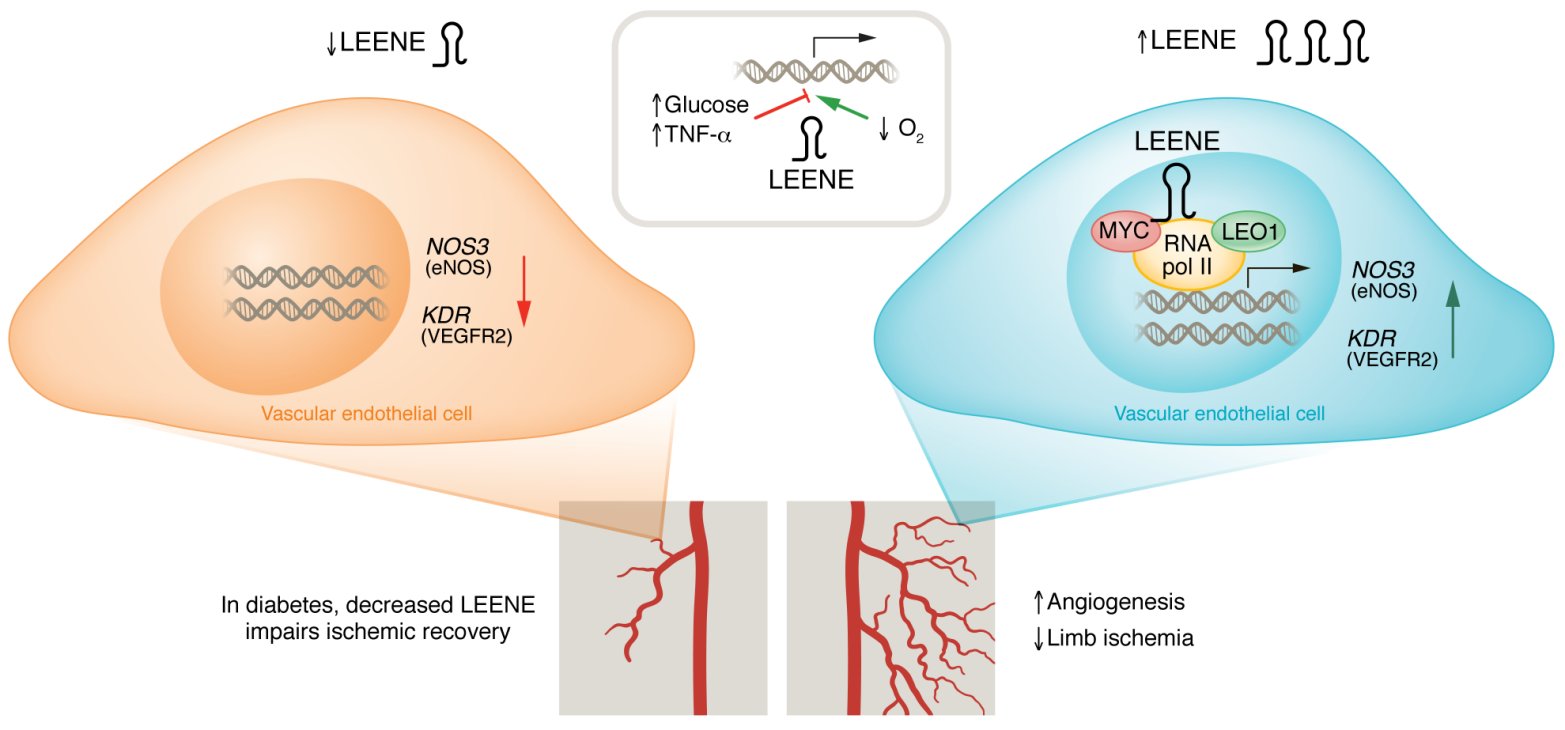
LEENE induces angiogenesis in peripheral arterial disease. Metabolic disruption, such that which occurs in diabetes, often results in impaired angiogenesis, and patients with diabetes have increased risk for limb ischemia. LEENE is downregulated by hyperglycemia and inflammation and upregulated by hypoxia. Loss of LEENE decreases angiogenesis and increases ischemic recovery time. In contrast, increasing LEENE levels improves these defects despite diabetic conditions, providing a target for peripheral arterial disease.

Several studies have shown that lncRNAs participate in vascular pathogenesis (6, 7). In this issue of the *JCI*, Tang, Luo, et al. (8) expand on the interweaving of vascular biology with noncoding gene regulation. The authors found that the lncRNA that enhances endothelial nitric oxide synthase (eNOS) expression (abbreviated as LEENE) was essential for angiogenesis and maintenance of tissue perfusion. Previously, the same group had identified LEENE as an enhancer-derived lncRNA that regulates eNOS expression in endothelial cells (9). Since metabolic perturbations such as diabetes can lead to an impaired angiogenic response (10), the authors leveraged RNA-seq data to identify factors divergently regulated with proangiogenic stimuli and glucose signaling. LEENE was induced in murine and human endothelial cells in response to hypoxia. In contrast, it was downregulated during hyperglycemia and inflammation. In line with these results, a high-fat diet in murine models led to LEENE downregulation, suggesting that LEENE is tightly regulated in response to environmental cues. The authors used LNA gapmers to test the contributions of LEENE to an angiogenic response in vitro. Loss of LEENE led to a reduction in key proangiogenic factors, including eNOS, VEGFR2, and placental growth factor (PGF). In addition, loss of LEENE in human endothelial cells led to a reduction in functional measures of angiogenesis, such as tube formation, sprouting capacity, and wound closure (Figure 1).

Arguably, one of the most substantial conceptual advances of Tang, Luo, et al. (8) was the development of a lncRNA knockout (KO). Since lncRNAs can have highly context-specific functions, their effects may be subtle or compensated in vivo. Thus, KO models are crucial in elucidating the physiologic contributions of a given lncRNA. Despite a growing number of lncRNA-KO models, few models have been generated in cardiovascular biology (11). Thus, generation of *Leene*-KO mice represents a welcome advance. Genetic deletion of *Leene* was not associated gross abnormalities in weight, blood pressure, viability, or development. However, in a critical limb-ischemia model under conditions that mimic hyperglycemia, *Leene*-KO mice had impaired flow recovery and lower microvascular density. Thus, the mouse model suggests that LEENE is essential for ischemic angiogenic responses under metabolic stress (Figure 1).

A common challenge in lncRNA studies involves disentangling the functional contributions of the RNA from the encoded genomic elements at the same locus (12). In other words, does the phenotype of the *Leene* KO depend on the RNA transcript or on the underlying DNA, which may be involved in *cis*/long-range interactions? To address this question, Tang, Luo, and colleagues (8) expressed the *LEENE* transcript, or a control gene, in *Leene*-KO mice, showing that expression of LEENE was sufficient to rescue the perfusion and capillary density defect. These findings suggest that, at least in part, *Leene* RNA operates in *trans* and can promote angiogenesis independent of its cognate DNA. Most lncRNAs lack clear evidence suggestive of cross-species relevance, owing to the minimal sequence conservation of most lncRNAs. The studies presented by Tang, Luo, and colleagues (8), however, suggest that LEENE is functionally conserved, since the human ortholog was used in the in vivo rescue experiments.

## Using RNA ChIP to decipher lncRNA mechanisms

LEENE is predominantly present in the nucleus in association with chromatin. A number of techniques have been developed to interrogate the binding patterns of chromatin-associated lncRNA. Examples include chromatin isolation by RNA purification (ChIRP), RNA antisense purification (RAP), and capture hybridization analysis of RNA targets (CHART) (13). Analogous to chromatin immunoprecipitation and sequencing (ChIP-seq) for transcription factors, the ChIRP technique uses a series of tiling oligonucleotides that allow genome-wide mapping of RNA contact sites. In addition, when coupled with mass spectrometry (MS), ChIRP-MS can directly interrogate protein binding partners (14). To map LEENE DNA-binding sites, the authors performed ChIRP-seq and showed that LEENE RNA bound angiogenic gene promoter regions such as *NOS3* (eNOS) and *KDR* (VEGFR2) loci. This binding was RNA dependent since it was abolished in control experiments incorporating RNase. In addition, ChIRP-MS reveled that LEENE interacted with LEO1, a key subunit of the RNA polymerase II–associated factor complex (Paf1C). Importantly, knockdown of LEO1 abolished LEENE-induced expression of angiogenic factors like eNOS and VEGFR2. MYC, a known binding partner of LEO1, also directly interacted with LEENE and these findings are consistent with previous work showing a role for MYC in direct regulation of *KDR* and angiogenesis (15, 16). Thus, in their model, the authors propose that LEENE promotes the biogenesis of proangiogenic factors by associating with transcription factors LEO1 and MYC.

## Implications and future directions

The field of lncRNAs has come a long way from the selfish genetic elements narrative of the 1980s. The characterization of LEENE adds to the physiologic contributions of lncRNAs in health and disease. It is known that patients with diabetes are at substantially higher risk for limb ischemia even when correcting for other covariates (17). The fact that LEENE, a proangiogenic lncRNA, is potently suppressed during hyperglycemia suggests that it may contribute to the impaired flow and angiogenic response seen in patients with metabolic diseases. One can speculate that approaches that would enhance LEENE expression (for example, RNA mimetics) could mitigate the effects of peripheral vascular disease in diabetes. Given their highly context-specific effects and expression patterns, lncRNAs have been proposed as promising disease biomarkers and therapeutic targets. Unfortunately, these theoretical aspirations have not materialized and most lncRNA translational studies have not led to substantial progress in clinical practice. It should also be noted that the effect of LEENE perturbation in mice was not dramatic, albeit significant (*P* values < 0.05), and therefore, future work will determine the durability of the findings reported here in other models and their potential therapeutic implications.

Few studies in cardiovascular disease have leveraged RNA-centric chromatin assays to decipher lncRNA mechanisms. These studies are known to be highly tedious and require extensive optimization, but have the advantage of being unbiased. Capitalizing on this approach, the authors identified LEENE target genes and interacting partners, which integrates well with the proposed function of LEENE. These exciting studies, however, raise a number of important questions. First, the Achilles heel of most lncRNA studies can be summarized in one word: stoichiometry. lncRNAs are often much less abundant than their interacting partners. There are examples that explain how lncRNAs may regulate highly abundant proteins. For instance, the lncRNA NORAD regulates the much more abundant Pumilio protein by inducing its phase separation (18). Thus, key inquiries moving forward involve understanding the biochemical basis of LEENE interactions with highly abundant proteins like MYC and LEO1. Further, the likely preservation of LEENE activity across species provides a unique opportunity to understand the structural basis for LEENE functional conservation. What domains of LEENE are crucial for its chromatin and protein interactions? Does LEENE impact only a subset of MYC and LEO1 targets? If so, what is the molecular basis for this specificity? Are there other functions for LEENE that may be independent of its effects in endothelial cells? We eagerly await future studies that answer these questions and others.

## Version 1 02/01/2023

Electronic publication
